# Positive association of the hepatic lipase gene polymorphism c.514C > T with estrogen replacement therapy response

**DOI:** 10.1186/1476-511X-10-197

**Published:** 2011-11-02

**Authors:** Alvaro Pulchinelli, Ana Maria Massad Costa, Cristina V de Carvalho, Naiara Correa Nogueira de Souza, Mauro A Haidar, Adagmar Andriolo, Ismael DC Guerreiro da Silva

**Affiliations:** 1 Laboratório de Biologia Molecular, Departamento de Ginecologia, Escola Paulista de Medicina, Universidade Federal de São Paulo, São Paulo, SP, Brasil; 2 Setor de Climatério, Departamento de Ginecologia, Escola Paulista de Medicina, Universidade Federal de São Paulo, São Paulo, SP, Brasil; 3 Setor de Patologia Clínica/Medicina Laboratorial, Departamento de Medicina, Escola Paulista de Medicina, Universidade Federal de São Paulo, São Paulo, SP, Brasil

**Keywords:** Hepatic lipase gene, Menopause, Hormone replacement therapy, Lipid metabolism, SNP

## Abstract

**Background:**

Hepatic lipase (HL), an enzyme present in the hepatic sinusoids, is responsible for the lipolysis of lipoproteins. Human HL contains four polymorphic sites: G-250A, T-710C, A-763G, and C-514T single-nucleotide polymorphism (SNPs). The last polymorphism is the focus of the current study. The genotypes associated with the C-514T polymorphism are CC (normal homozygous - W), CT (heterozygous - H), and TT (minor-allele homozygous - M). HL activity is significantly impaired in individuals of the TT and CT genotypes. A total of 58 post-menopausal women were studied. The subjects were hysterectomized women receiving hormone replacement therapy consisting of 0.625 mg of conjugated equine estrogen once a day. The inclusion criteria were menopause of up to three years and normal blood tests, radiographs, cervical-vaginal cytology, and densitometry. DNA was extracted from the buccal and blood cells of all 58 patients using a commercially available kit (GFX^® ^- Amersham-Pharmacia, USA).

**Results:**

Statistically significant reductions in triglycerides (t = 2.16; n = 58; p = 0.03) but not in total cholesterol (t = 0.14; n = 58; p = 0.89) were found after treatment. This group of good responders were carriers of the T allele; the CT and TT genotypes were present significantly more frequently than in the group of non-responders (p = 0.02 or p = 0.07, respectively). However, no significant difference in HDL-C (t = 0.94; n = 58; p = 0.35) or LDL-C (t = -0.83; n = 58; p = 0.41) was found in these patients.

**Conclusions:**

The variation in lipid profile associated with the C-514T polymorphism is significant, and the T allele is associated with the best response to ERT.

## Background

Hepatic lipase (HL), an enzyme present in the hepatic sinusoids, is responsible for the lipolysis of lipoproteins. Although also expressed in other tissues, over 95% percent of the total HL activity is found in the liver [1]. As a key enzyme in lipoprotein metabolism, HL hydrolyzes triglycerides (TG), intermediate-density lipoprotein (IDL-C), high-density lipoprotein (HDL-C), and the surface phospholipids and core TG of small very-low-density lipoprotein (VLDL-C) remnants. HL also aids in the binding of lipoproteins to cellular receptors. In vitro studies have demonstrated that HL facilitates the binding and entrance of chylomicrons, chylomicron remnants, VLDL-cholesterol (VLDL-C), LDL-C [2,3] and HDL cholesterol (HDL-C) [4-6] into a number of different cell types. Fertile females have lower HL activity than males [7,8].

Patients with HL deficiency often present with hypercholesterolemia, hypertriglyceridemia and increased levels of VLDL-C, remnant chylomicrons, IDL-C, TG-rich LDL-C, and HDL-C [9-11]. However, because not all patients with HL deficiency show these metabolic changes, the condition is sometimes confused with other genetic disorders [12].

The human HL gene, a 60 kb gene with a 1.6 kb exon, is present on chromosome 15 [13,14]. The gene possesses four known polymorphic sites, and these four sites are in complete linkage disequilibrium. The most common polymorphisms are G-250A, T-710C, A-763G and C-514T [7], and the most common haplotypes are GTAC and ACGT. Because the C-514T polymorphism occurs in the promoter region of the HL gene, it has been suggested that this polymorphism may influence the metabolic activity of the enzyme [15-17] but not influence transcription [18]. Some authors have found that the polymorphisms do not cause phenotypic changes in females, although they have noted a HDL-C difference in men [18,19]. Because HL activity may also be controlled by circulating estrogen [16], it is likely that further studies of the HL gene polymorphisms will provide additional genetic information that may be useful in developing more successful hormone replacement therapies [20].

A meta-analysis of 24,000 cases has demonstrated that HL activity is significantly lower in individuals with the TT and CT genotypes for the C-514T polymorphism than in individuals with the CC genotype [20]. Despite its association with high HDL cholesterol, the presence of the T allele results in a slightly increased risk of atherosclerosis [7]. Human and animal studies have found both atherogenic and anti-atherogenic activities for the enzyme [21]. While low HL activity is associated with a greater risk of arterial coronary disease (ACD) [22], it is not directly responsible for this disorder [23]. Although there seems to be an inverse relationship between HL level and atherosclerotic disease, a consensus is lacking on this point; some studies have found increased HL in patients with ACD [24]. In humans, increased HL is associated with small, dense LDL-C particles and with lower levels of antiatherogenic large HDL-C particles [24,25]. However, studies using animal models have not confirmed this relationship, which suggests that other factors may contribute to the development of ACD. Because HL hydrolyzes phospholipids and triglycerides and releases Fatty Acids (FA) from phospholipids and triglycerides [26], increased HL expression may cause more cholesterol to enter cells and sub-endothelial LDL-C deposits, thus promoting phagocytosis by macrophages and increasing the risk of atherosclerotic lesions. As has been mentioned, HL can have both pro- and anti-atherogenic effects. As an enzymatic or binding protein, it can change the level, composition or metabolism of lipoproteins in ways that can either increase or decrease atherogenesis. In the presence of hypertriglyceridemia and increased LDL-C, high HL activity (the formation of small, dense LDL-C) has a pro-atherogenic effect. However, low levels of LDL-C and high levels of HL may be due to the anti-atherogenic effect of increased VLDL-C remnants and IDL-C catabolism [27-29]. The pro- and anti-atherogenic effects of HL may be mediated by the formation of increased levels of small dense LDL-C in the presence of hypertriglyceridemia and by the increased catabolism of VLDL-C remnants and IDL-C, respectively. A number of researchers believe that HL plays an important role in the reverse transport of cholesterol and that its role in this process may be the primary mechanism responsible for the development of atherosclerotic disease [30-34]. There is still great controversy in the literature regarding the pro- and anti-atherogenic roles of HL [32].

Findings that suggest that high HL is responsible for increased atherogenic potential include smaller LDL-C particle size with increased HL activity, enzyme activity that is inversely proportional to HDL-C level, greater activity in males than in fertile females, and a positive correlation of HL level with insulin resistance and/or abdominal fat. Two findings that support an anti-atherogenic role for the enzyme include its reverse cholesterol transport activity and its role in TG clearing after meals [35]. Environmental factors must also be taken into account. There may be a strong interaction effect of HL polymorphisms and the amount and type of dietary fat on HDL-C level [36]. Body mass index (BMI), or adiposity, also appears to affect HL activity, and this effect is additive with the effects of the genetic polymorphisms that have been analyzed [37]. There is also evidence that HL activity is specifically related to the amount of intra-abdominal fat [17,38].

A number of recent studies have failed to support the efficacy of hormone replacement therapy [39-42]. The Heart and Estrogen/Progestin Replacement Study (HERS) conducted by Hulley in 1998 did not recommend the use of HRT for the secondary prevention of arterial coronary disease (ACD); however, due to good ACD outcomes in women taking HRT for several years, HRT continues to be used [39]. A recent study has found that taking estrogen for a mean of 5.9 years was not associated with any change in a woman's ACD risk [43]. Other studies have suggested that hormone replacement therapy may cause pro-thrombotic susceptibility in women with prior atherosclerosis [44].

Estrogen increases plasma levels of apolipoprotein A-1 (ApoA-1) and HDL-C, possibly by increasing ApoA-1 expression in the liver, and it modulates the expression of proteins involved in HDL-C metabolism. Thus, we can expect a high degree of inter-individual variability in HRT response [45]. In rats, estrogen also seems to be associated with the modulation of HL gene expression, and raising HL level decreases HDL-C level [15]. The mechanism by which estrogen reduces LDL-C and increases HDL-C is different from the mechanism of lipid-lowering drugs. This difference may explain why HRT is not effective in preventing the progression of coronary heart disease in postmenopausal women [46,47]. HL activity is susceptible to variations in endogenous sexual steroids. Estrogenic steroids suppress HL activity, while androgenic steroids increase it [8,48]. The effect of the C514T polymorphism on HL activity is independent of androgen action [49].

The current study was motivated by the controversy regarding the pro- and anti-atherosclerotic effects of HL in women undergoing estrogen replacement therapy. The objective of the study was to optimize hormone replacement therapy by establishing the genetic patterns associated with better responses to such treatment.

## Results

### Comparative analysis before and after treatment

The results are presented as the mean and standard deviation of the variables (Table 1). Table 1 shows the influence of estrogen replacement treatment on the group as a whole. The values for cholesterol, HDL-C, LDL-C, and triglycerides were analyzed independently of genotype for the both pre- and post-treatment conditions.

**Table 1:** Comparative analysis of the variables before and after treatment, independent of genotype

Groups	N	Mean (mg/dL)	Standard deviation	t	*p*
TotalChol	58	210.31	45.47	0.14	**0.89**
		
TotalChol2	58	209.41	43.19		
		
HDL-C	58	58.95	13.45	0.94	**0.35**
		
HDL-C2	58	57.76	14.44		
		
LDL-C	58	120.052	38.71	-0.83	**0.41**
		
LDL-C2	58	125.52	38.08		
		
TG	58	138.07	65.40	2.16	**0.03**
		
TG2	58	122.07	66.50		

TotalChol: total pre-treatment cholesterol; HDL-C: pre-treatment high-density lipoprotein cholesterol; LDL-C: pre-treatment low-density lipoprotein cholesterol; TG: pre-treatment triglycerides; TotalChol2: total post-treatment cholesterol; HDL-C2: post-treatment high-density lipoprotein cholesterol; LDL-C2: post-treatment low-density lipoprotein cholesterol; TG2: post-treatment triglycerides.

All of the genotypes were in Hardy-Weinberg equilibrium (Table 2).

**Table 2:** Numbers of expected and observed genotypes according to Hardy-Weinberg equilibrium

	CC (N)	CT (N)	TT (N)	Chi-Square	p*
Observed	15	29	14	0.000005	0.998

Expected	15.00	28.99	14.00		

CC (major-allele homozygote); CT (heterozygote); TT (minor-allele homozygote)

*p= 0,998

### Comparative analysis of different genotypes before and after treatment

To evaluate whether the baseline values of any of the variables were influenced by genotype, Table 3 presents the lipid profiles by genotype (major-allele homozygote, heterozygote, and minor-allele homozygote).

**Table 3:** Influence of genotype on baseline lipid levels

Genotype	Variable	N	Mean	Standard Deviation	t-test	p
CC	TotalChol	15	216.27	47.36	0.15	0.89

CT	TotalChol	29	205.42	44.03	0.08	0.94

TT	TotalChol	14	214.07	48.65	0.03	0.98

CC	HDL-C	15	57.87	12.56	1.31	0.21

CT	HDL-C	29	57.17	12.18	0.21	0.84

TT	HDL-C	14	63.78	16.44	0.36	0.72

CC	LDL-C	15	134.40	42.83	-0.09	0.93

CT	LDL-C	29	118.21	31.19	-0.14	0.89

TT	LDL-C	14	108.50	45.96	-1.26	0.23

CC	TG	15	132.60	45.50	1.07	0.30

CT	TG	29	136.41	70.19	1.20	0.24

TT	TG	14	147.36	76.01	1.71	0.11

TotalChol: total pre-treatment cholesterol; HDL-C: pre-treatment high-density lipoprotein cholesterol; LDL-C: pre-treatment low-density lipoprotein cholesterol; TG: pre-treatment triglycerides.

To further study this potential influence, Table 4 compares the means of the variables by genotype (shows the results of testing). For this purpose, we used an ANOVA model and compared the means by group.

**Table 4:** An ANOVA model comparing the mean baseline lipid levels by genotype (major-allele homozygote, heterozygote, and minor-allele homozygote)

ANOVA						
		Sum of squares	df	Mean square	F	Sig
TotalChol	Between Groups	1425.517	2	712.759	0.337	0.716
		
	Within Groups	116446.9	55	2117.216		
		
	Total	117872.4	57			

HDL-C	Between Groups	436.616	2	218.308	1.216	0.304
		
	Within Groups	9874.228	55	179.531		
		
	Total	10310.845	57			

LDL-C	Between Groups	5054.986	2	2,527.493	1.73	0.187
		
	Within Groups	80373.859	55	1461.343		
		
	Total	85428.845	57			

TG	Between Groups	1735.875	2	867.938	0.197	0.822
		
	Within Groups	242037.8	55	4400.688		
		
	Total	243773.7	57			

We also analyzed the lipid levels before and after ERT; these results are shown according to genotype in Table 5. In this analysis, we studied the influence of the treatment on lipid levels. Table 6 shows the influence of the T allele on the lipid profile and response to estrogen treatment.

**Table 5:** Results of the paired-samples t-tests (before and after treatment) according to genotype

Genotype	Variable (mg/dL)	N	Mean	Standard Deviation	t-test	*p*
CC	TotalChol	15	216.27	47.36	0.15	0.89
			
	TotalChol 2	15	214.60	39.09		

CT	TotalChol	29	205.41	44.03	0.08	0.94
			
	TotalChol 2	29	204.66	39.76		

TT	TotalChol	14	214.07	48.65	0.03	0.98
			
	TotalChol 2	14	213.71	55.03		

CC	HDL-C	15	57.87	12.56	1.31	0.21
			
	HDL-C 2	15	54.87	15.47		

CT	HDL-C	29	57.17	12.18	0.21	0.84
			
	HDL-C 2	29	56.76	13.38		

TT	HDL-C	14	63.79	16.44	0.36	0.72
			
	HDL-C 2	14	62.93	15.16		

CC	LDL-C	15	134.40	42.83	-0.09	0.93
			
	LDL-C2	15	135.40	34.27		

CT	LDL-C	29	118.21	31.19	-0.14	0.89
			
	LDL-C2	29	19.62	38.01		

TT	LDL-C	14	108.50	45.96	-1.26	0.23
			
	LDL-C2	14	127.14	42.28		

CC	TG	15	132.60	45.50	1.07	0.30
			
	TG2	15	121.13	44.02		

CT	TG	29	136.41	70.19	1.20	0.24
			
	TG2	29	121.83	73.78		

TT	TG	14	147.36	76.01	1.71	0.11
			
	TG2	14	123.57	74.68		

TotalChol: total pre-treatment cholesterol; HDL-C: pre-treatment high-density lipoprotein cholesterol; LDL-C: pre-treatment low-density lipoprotein cholesterol; TG: pre-treatment triglycerides; TotalChol2: total post-treatment cholesterol; HDL-C2: post-treatment high-density lipoprotein cholesterol; LDL-C2: post-treatment low-density lipoprotein cholesterol; TG2: post-treatment triglycerides; CC: homozygous major-allele genotype; CT: heterozygous genotype; TT: homozygous minor-allele genotype.

**Table 6:** Paired-samples t-tests (before and after treatment) for the CT and TT genotypes

(mg/dL)		Paired differences					t	df	Sig (2-tailed)
					
		Mean	SD	Mean Standard Error	95% Confidence interval				
								
					Lower	Upper			
Pair 1	TotalChol -TotalChol2	0.62791	50.96685	7.77237	-15.05738	16.31319	0.081	42	0.936

Pair 2	HDL-C - HDL-C-C2	0.55814	9.9506	1.51745	-2.5042	3.62048	0.368	42	0.715

Pair 3	LDL-C - LDL-C2	7.02326	53.53614	8.16419	-23.49925	9.45274	-0.86	42	0.395

HDL: pre-treatment high-density lipoprotein; LDL: pre-treatment low-density lipoprotein; VLDL: pre-treatment very-low-density lipoprotein; HDL2: post-treatment high-density lipoprotein; LDL2: post-treatment low-density lipoprotein

No statistically significant differences in total cholesterol, HDL-C, or LDL-C were found after re-grouping the genotypes into CT and TT (Table 6). Table 6 shows the influence the T allele on the lipid profile and estrogen treatment.

Table 7 summarizes the data and shows the correlations between the C and T alleles and the baseline lipid values. An ANOVA model was used to compare the mean lipoprotein levels in the patients with C and T alleles.

**Table 7:** An ANOVA model comparing the baseline means of the lipid variables between the C and T alleles

ANOVA						
		Sum of squares	df	Mean square	F	Sig
TotalChol	Between Groups	46.432	1	46.432	0.022	0.881
		
	Within Groups	235698.4	114	2067.53		
		
	Total	235744.8	115			

HDL-C	Between Groups	243.083	1	243.083	1.36	0.246
		
	Within Groups	20378.607	114	178.76		
		
	Total	20621.69	115			

LDL-C	Between Groups	4901.112	1	4901.112	3.367	0.069
		
	Within Groups	165956.6	114	1455.759		
		
	Total	170857.7	115			

TG	Between Groups	1551.263	1	1551.263	0.364	0.548
		
	Within Groups	485996.2	114	4263.124		
		
	Total	487547.4	115			

Table 8 shows the influence of the C and T alleles on the lipoprotein levels of the patients receiving hormone replacement with estrogen. A multivariate ANOVA model was used to compare the means of the patients with the C and T alleles. We then compared the lipoprotein changes in response to the treatment, according to genotype.

**Table 8:** Results of the paired-samples t-tests for lipoproteins before and after treatment, by allele

Allele	Variable mg/dL	N	Mean	SD	t-test	p
C	TotalChol	59	210.93	45.26	0.20	0.85
			
	TotalChol2	59	209.71	39.06		

T	TotalChol	57	209.67	45.68	0.08	0.93
			
	TotalChol2	57	209.11	47.08		
			

C	HDL-C	59	57.53	12.16	1.37	0.18
			
	HDL-C2	59	55.80	14.24		

T	HDL-C	57	60.42	14.52	0.50	0.62
			
	HDL-C2	57	59.79	14.35		

C	LDL-C	59	126.44	37.70	-0.20	0.84
			
	LDL-C2	59	127.44	36.44		

T	LDL-C	57	113.44	38.62	-1.39	0.17
			
	LDL-C2	57	123.32	39.58		

C	TG	59	134.47	58.15	1.85	**0.07**
			
	TG2	59	121.47	59.69		

T	TG	57	141.79	71.95	2.46	**0.02**
			
	TG2	57	122.68	72.88		

TotalChol: total pre-treatment cholesterol; HDL-C: pre-treatment high-density lipoprotein cholesterol; LDL-C: pre-treatment low-density lipoprotein cholesterol; TG: pre-treatment triglycerides; TotalChol2: total post-treatment cholesterol; HDL-C2: post-treatment high-density lipoprotein cholesterol; LDL-C2: post-treatment low-density lipoprotein cholesterol; TG2: post-treatment triglycerides; CC: homozygous major-allele genotype; CT: heterozygous genotype; TT: homozygous minor-allele genotype.

## Discussion

A statistically significant difference in triglyceride level was observed after ERT compared to baseline (Table 1). To examine the efficacy of ERT, we did not differentiate the subjects by genotype. No statistically significant differences in lipoprotein levels were observed. The p values for lipoprotein levels before and after treatment were p = 0.89 and p = 0.35 for HDL-C and LDL-C, respectively; in other words, HDL-C and LDL-C levels did not change significantly after treatment. These findings are probably related to the fact that the major substrates of HL are TG and HDL-C rather than LDL-C [17,26,27].

No statistically significant differences in the baseline level of total cholesterol, HDL-C, LDL-C, or triglycerides were observed among patients of different genotypes (Tables 3 and 4). This finding is interesting because the current literature suggests that polymorphisms can directly influence HL activity, as revealed by differences in lipid levels [48]. Recent results are consistent with our findings regarding total cholesterol [18]. Our findings were obtained using an ANOVA model that compared the baseline means of the three different groups (CC, CT and TT). The T allele was associated with higher HDL-C in those individuals with a higher proportion of fat in their diets. No significant differences were observed among CC and CT individuals [36]. Because the patients in this study did not follow a specific diet, we believe that potential differences may have been overshadowed by the variation in the patients' dietary habits. This factor may account for the published data showing associations of the 514C > T polymorphism with measures of HDL-C metabolism, depending on the amount and type of fat consumed [36].

When the three genotypes were analyzed separately, no significant changes in the triglycerides following ERT were observed. Moreover, this reduction was primarily observed in T allele carriers (Table 5). To explore the hypothesis that the T allele may have influenced the results, the genotypes were re-classified as either CT or TT. Although a reduction in lipoprotein was observed, it was not statistically significant (t = 1.889, p = 0.06).

The significant decrease in triglycerides observed in the first analysis was seen only in the CT (t = 1.20; p = 0.24) and TT (t = 1.71; p = 0.11) genotypes. A significant decrease in triglycerides (t = 3.22; p = 0.02) in T carriers was also found when the CT and TT genotypes were grouped together. In contrast, those with the CC genotype did not show a significant decrease in triglycerides after the treatment (t = 1.07; p = 0.30) [50].

HDL-C and LDL-C did not differ among genotypes either before or after treatment, even after dividing analyzing the CT and TT genotypes separately (Table 6). All of the two-tailed *p *values associated with these comparisons were greater than 0.05. There is speculation about the relationships between HDL-C metabolism and HL polymorphisms. However, these polymorphisms result in only minor variations in HDL-C levels [51], which is consistent with our findings.

We next examined the influence of the C and T alleles on the baseline values and on the pre- and post-treatment conditions. No statistically significant differences in any of the baseline variables were observed between the patients with the C allele and those with the T allele (Tables 6 and 7). This finding suggests that the presence of the T allele (and low hepatic lipase activity) may be associated with a slightly increased risk of atherosclerosis, despite the high HDL cholesterol in individuals with the T allele.

The T allele was associated with a significant reduction in triglycerides. Reduced triglycerides after ERT were observed in individuals of both the TT and CT genotypes, but the reduction was more significant in T than C alleles. These results are indirectly expressed with respect to genotype; the comparison of the CT and TT patients showed a trend toward a similar level of triglyceride reduction (t = 1.889; p = 0.06) (Table 8). These findings indicate that the mechanism of HRT differs from that of the typical lipid-lowering drugs; if the mechanism were the same, the treatment would be expected to have a greater effect on those with the C allele [7,8,50].

The limitation of our study is the relatively small number of patients included. However, a strength of our study is that it is a prospective study of the Brazilian population, comparing the same women before and after HRT to address the question of whether or not lipid levels are associated with the alleles C or T.

## Conclusions

In conclusion, HRT seems to reduce triglyceride levels regardless of the genotype at the C-514T polymorphic locus. Although individuals with the T allele who receive HRT appear to have a positive lipid profile, the antiatherogenic effect of the T allele is not clear [52,53]. However, the T allele (p-values = 0.93 and 0.02 for cholesterol and triglycerides, respectively) does seem to be a better genetic marker for total cholesterol and triglyceride levels than the C allele (*p *values = 0.85 and 0.07 for cholesterol and triglycerides, respectively). According to the literature [47], there is large inter-individual variability in the plasma lipid response to HT. A similar effect was not observed for LDL-C or HDL-C.

## Methods

### Subjects

The study population was derived from a larger study of hormone replacement therapy in postmenopausal women. The larger study included a total of 200 patients from a tertiary outpatient clinic. One hundred of these women had undergone hysterectomy for myomas and were receiving HRT consisting of a single daily dose (0.625 mg) of conjugated equine estrogen The 100 non-hysterectomized women in the study received hormone replacement therapy consisting of a daily combination of 0.625 mg estrogen and 2.5 mg of medroxyprogesterone (estrogen and progestin replacement therapy). Only those patients who received conjugated equine ERT exclusively and who presented lipid profiles within the reference range were enrolled in our study. A total of 58 patients with a mean age of 54 years were selected for DNA extraction and the subsequent study of the C-514T HL gene polymorphism both before and after ERT.

The inclusion criteria included the following: 1) a maximum three-year history of menopause; 2) complete chart information with annual blood work (complete blood count with differential, total cholesterol and fractions, triglycerides, urea, creatinine, blood glucose, estradiol, progestin, FSH, and LH) that was within the reference range since the onset of menopause; 3) a bilateral mammogram with BI-RADS classification 1 or 2; 4) abdominal and pelvic ultrasounds with normal findings; 5) cervical-vaginal cytology that was normal by the Bethesda classification; and 6) bone densitometry (patients within one standard deviation of the age-adjusted mean).

Women who did not comply with the proposed hormone replacement therapy were excluded from the study, as were those who did not meet the above criteria.

Information on alcohol use and other lifestyle factors, such as physical activity and dietary saturated fat, was not collected. The patients were not instructed to follow any specific dietary guidelines.

All patients gave free and informed consent to participate, and the project was approved by the Committee of Ethics in Research at the Federal University of São Paulo, with the number *CEP 1114/08*

## Methods

Buccal tissue samples were obtained with a cytobrush. Samples were placed in Falcon tubes with Tris-EDTA buffer and frozen at -80°C for subsequent genomic DNA extraction. Blood samples were obtained before and after ERT using a Vacutainer^®^. The patients were instructed how to prepare for sampling so as to minimize interference from pre-analytical factors. The patients were instructed to fast from 10 to 14 hours, to maintain their usual diet, not to use alcohol within 72 hours prior to the collection and not to perform strenuous exercise on the collection day [54]. Total cholesterol was measured using the oxidase method. HDL-C was measured by the direct method and by a homogenous assay. Triglycerides were measured using an enzymatic colorimetric method (Advia 1650; Bayer^®^, USA). The LDL-C concentration was obtained using Friedwald's formula [55]. The intra-assay coefficients of variability were less than 5%.

DNA of the buccal mucosa was analyzed using a commercially available PCR kit (GFX^®^; Amersham-Pharmacia, USA) according to the manufacturer's instructions. PCR was carried out using primers to detect the C-T change, as described by Somekawa et al. [56]. The following primers were used in this study: LipR, 5'-TCACTTGGCAAGGGCATCTTTG-3'; and LipF, 5'-GGTCGGGGTAGGTGGCTTCCA-3'. The PCR protocol included 35 cycles at 94°C for 30 s, 55°C for 60 s and 72°C for 90 s, after 2 min of denaturation at 95°C. PCR fragments were digested with 1.5 U of NiaIII restriction enzyme, followed by electrophoresis in a 3% low-melting agarose gel that was then stained with ethidium bromide. The size of the amplified fragment was 274 bp. Fragments of 226 and 48 bp were obtained for allele T, and fragments of 274 bp were obtained for allele C. The genotypes were expressed as CC (major-allele homozygote (W), 274 bp), CT (heterozygote (H), 274, 226, and 48 bp), and TT (minor-allele homozygote (M), 226 and 48 bp).

### Statistical analysis

Because each individual had more than one set of observations, we used the paired-samples t-test to compare the quantitative data; this test is equivalent to a standard Student's t-test but is specifically designed for related samples. It requires that the data have a normal distribution. Thus, we initially used the Kolmogorov-Smirnov test to verify that the data were normally distributed. The p value of the non-normality hypothesis was > 0.05 for all of the variables. The results are presented as mean and standard deviation.

To determine whether genotype influenced the baseline levels of the variables, we used analysis of variance (ANOVA). We compared the average values for the three genotypes in the samples taken before and after treatment with ERT. The variables included age, total cholesterol, HDL-C, LDL-C and TGL. Hardy-Weinberg equilibrium was checked for the entire sample.

## Abbreviations

ACD: arterial coronary disease; ANOVA: analysis of variance; ApoA-1: apolipoprotein A-1; BMI: body mass index; ERT: estrogen replacement therapy; FA: fatty acids; HRT: hormone replacement therapy; HL: hepatic lipase; HDL-C: high-density lipoprotein; IDL-C: intermediate-density lipoprotein; LDL-C: low-density lipoprotein; PCR: polymerase chain reaction; SNP: single-nucleotide polymorphism; TG: triglycerides; VLDL-C: very-low-density lipoprotein

## Competing interests

The authors declare that they have no competing interests.

## Authors' contributions

APJ: design and conduct of the study, data collection, data analysis, data interpretation and manuscript writing. AMMC: design and conduct of the study, data collection CVC: data interpretation. NNCS: data collection, data analysis and laboratory analysis in molecular biology MAH: data collection, data interpretation. AA: design of the study, data interpretation, manuscript writing. IDCGS: design and conduct of the study, data analysis, data interpretation, manuscript writing. All authors read and approved the final manuscript.
